# Lymph node ratio (LNR) as a complementary staging system to TNM staging in salivary gland cancer

**DOI:** 10.1007/s00405-019-05597-0

**Published:** 2019-09-11

**Authors:** Bo-Wen Lei, Jia-Qian Hu, Peng-Cheng Yu, Yu-Long Wang, Wen-Jun Wei, Ji Zhu, Xiao Shi, Ning Qu, Zhong-Wu Lu, Qing-Hai Ji

**Affiliations:** 1 Department of Head and Neck Surgery, Fudan University Shanghai Cancer Center, 270 Dong’an Road, Shanghai, 200032 China; 2 Department of Oncology, Shanghai Medical College, Fudan University, Shanghai, 200032 China; 3 Department of Radiation Oncology, Fudan University Shanghai Cancer Center, Shanghai, 200032 China; 4 Department of Statistics, Fudan University Shanghai Cancer Center, Shanghai, 200032 China

**Keywords:** Salivary gland cancer, Prognosis, TNM staging, Lymph node ratio, Radiotherapy

## Abstract

**Purpose:**

The role of lymph node ratio (LNR, ratio of metastatic to examined nodes) in the staging of multiple human malignancies has been reported. We aim to evaluate its value in salivary gland cancer (SGC).

**Methods:**

Records of SGC patients from Surveillance, Epidemiology, and End Results database (SEER, training set, *N* = 4262) and Fudan University Shanghai Cancer Center (FUSCC, validating set, *N* = 154) were analyzed for the prognostic value of LNR. Kaplan–Meier survival estimates, the Log-rank *χ*^2^ test and Cox proportional hazards model were used for univariate and multivariate analysis. Optimal LNR cutoff points were identified by X-tile.

**Results:**

Optimal LNR cutoff points classified patients into four risk groups, R0, R1 (≤ 0.17), R2 (0.17–0.56) and R3 (> 0.56), corresponding to 5-year cause-specific survival in SEER patients of 88.6%, 57.2%, 53.1% and 39.7%, disease-free survival in FUSCC patients of 69.2%, 63.3%, 34.6% and 0%, and disease-specific survival in FUSCC patients of 92.3%, 90.0%, 71.4% and 0%, respectively. Compared with TNM staging, TNM + R staging showed smaller AIC values and higher *C*-index values in the Cox regression model in both patient sets.

**Conclusions:**

LNR classification should be considered as a complementary system to TNM staging and LNR classification based clinical trials deserve further research.

**Electronic supplementary material:**

The online version of this article (10.1007/s00405-019-05597-0) contains supplementary material, which is available to authorized users.

## Introduction

Salivary gland cancer (SGC) accounts for 7–12% of head and neck cancers and has an increasing incidence [1–3]. Lymph node (LN) involvement is among the most important prognostic factors in SGCs [3–6]. Patients with pathological lymph node metastasis (pN+) are recommended for postoperative radiotherapy according to the National Comprehensive Cancer Network (NCCN) guideline [7]. Based on the number, laterality and size of metastasized lymph nodes, they are classified as N1–N3 according to the American Joint Committee on Cancer (AJCC) staging system [7].

To improve the prognostic system, one would intuitively take not only information on positive LNs but also the number of LNs examined (LNE) into account. Lymph node ratio (LNR), defined as the number of involved nodes divided by LNE, was found to improve prognostic information in breast, gastric, colorectal, bladder and skin cancers [8–12]. The subsequent study showed that the LNR also improved the comparisons between institutions compared with AJCC N staging [13]. However, to date, there have been no reports using the LNR in the staging of SGCs.

This study examined whether patients with SGC can be classified into meaningful risk categories based on the LNR (R classification). A TRM staging system which substituted the N classification of the TNM staging system with R classification was also developed and compared with the TNM staging system only and the combined TNM and R staging system (TNM + R), to determine the potential clinical significance of the LNR.

## Patients and methods

### Patients

The datasets of two groups of patients were used in the current study. This research was approved by the institution’s ethics committee. The SEER (Surveillance, Epidemiology, and End Results Program, https://www.seer.cancer.gov) database has been widely used for the analysis of LNR staging in colon, breast and skin cancer [11, 14, 15]. For the analysis of LNR staging of SGCs, the study population consisted of patients with determined LNR and detailed N staging information. For the survival analysis, the SEER cause-specific survival (CSS) was analyzed. Deaths attributed to the cancer of interest are treated as events and deaths from other causes are treated as a censored observation. Cases with non-concordant N classification information and number of regional positive nodes were rejected. Finally, 4262 patients with detailed lymph node examination information were collected, of which 1210 patients were pN+ and all the TNM staging were re-checked according to 2010 AJCC staging system. The cases with unclassified T, M classification, grade and other variables were also enrolled in the analysis set to avoid losing information and select bias, and were defined as Tx, Mx and unknown group. To compare the TNM staging system and TRM staging system, only 3944 patients were retrieved for analysis because 318 patients were excluded for without sufficient information (Tx and Mx) to determine the AJCC 7th edition stage.

The validating patient set was derived from the Fudan University Shanghai Cancer Center (FUSCC) dataset [5, 6]. In total, 246 consecutive SGC patients underwent primary surgery at the Department of Head and Neck Surgery between January 1998 and January 2010. Anatomic compartment and level-based neck dissections were performed in 62.6% (154/246) of cases. To avoid bias caused by occult LN metastasis, only the 154 patients with neck dissection were enrolled in the current study. Pathologic examinations showed that 42.9% (66/154) of patients were pN+, which included 14 pN1 and 52 pN2 according to the AJCC staging system. Follow-up strategies for all patients include physical examination, neck ultrasound and head MRI. The intervals for follow-up visits are every 3 months during the first 2 years, every 6 months in the third year and annually thereafter. The 5-year disease-specific survival (DSS, SGC specific) and disease-free survival (DFS, no local recurrence and distant metastasis) were 82.7% and 55.5%, respectively.

### Statistical analysis

The analysis was performed in four stages. For every step of survival analysis, we used the Log-rank *χ*^2^ test to identify the factors associated with mortality, followed by a multivariate analysis using forward stepwise regression with a Cox proportional hazards model. The survival rate and curves were calculated using the Kaplan–Meier method. Harrell’s concordance index (*C* index) and the AIC (Akaike information criterion) value related to the Cox regression model were analyzed to compare the predictive ability and relative goodness-of-fit between regression models [16]. A smaller AIC value and a higher *C* index value indicated a more desirable model for predicting the outcome. A *P* value of 0.05 was considered statistically significant. All statistical analyses were carried out using SPSS software version 17.0 (SPSS Inc., Chicago, IL, USA) and R2.14.0 software with packages (Boot, MASS and Survival).

First, we evaluated the prognostic value of LNR as a continuous variable, adjusting for other covariates associated with CSS in 1210 SEER pN+ cases. The stability of the results was tested by a bootstrap procedure, which applies proportional hazards computations to full random samples with the replacement of the patients. We ran 10,000 iterations in this procedure.

In the second stage, we proceeded to determine the most appropriate cutoff points for categorizing LNR as high, medium, and low-risk groups. Optimal thresholds for LNR and the most appropriate method for deciding cutoff points differs among investigators [9, 10, 17]. In the current study, LNR modeling using spline smoothing functions was used to evaluate the effect of LNR on SEER CSS [17]. Two pairs of cutoff points were identified using different methods and compared with LNR as a continuous variable to identify the optimal cutoff points. The first pair of cutoff points were identified by tertiles to split the patients into equal-sized groups [17]. The second pair of cutoff points were calculated by X-tile using the minimum *P* values from Log-rank *χ*^2^ statistics [18].

In the third stage, to determine the clinical usefulness of LNR staging, 4262 patients with detailed lymph node staging information were classified as R0-3, a total number of four groups using identified cutoff points (R classification). Either N or R classification and variables associated with CSS were enrolled in the multivariate Cox regression analysis to compare the predictive ability of both classifications. The predictive accuracy of SEER CSS of the TNM, TRM and TNM + R staging system (both TNM staging and R classification were analyzed in the Cox model as two variables) were compared by enrolled individually in the Cox regression model with covariates associated with CSS in 3944 patients with detailed TNM staging information.

Finally, the prognostic significance of LNR staging was validated in FUSCC patients. Since SEER dataset lacking certain clinicopathologic characteristics of the tumor as lymphatic/vascular/extracapsular invasion and tumor size, etc., the survival predictive model is not good enough for validation in a new patients set. We then validated the LNR classification itself in the FUSCC set. N, R classification, TNM and TNM + R staging system were adjusted individually with all the variates associated with DFS and DSS of 154 FUSCC patients identified by Log-rank *χ*^2^ test to compare the predictive ability of different staging system.

## Results

### LNR as a prognostic factor of SGCs by univariate and multivariate analysis

From the SEER dataset, 1210 pN+ patients were analyzed for the prognostic significance of LNR, and the clinical details were presented in Table 1. Univariate analysis identified that primary sites, histologic subtype, grade, T, N, and M classification were all prognostic factors of SEER CSS (cause-specific survival, Supplement Table 1). The Cox regression model confirmed that primary sites, T, N, M classification, number of lymph nodes examined, LNR and age were significant prognostic factors of SGC SEER CSS (Table 2). Examinations of 10,000 bootstrap resamplings of the data with reiterations of the AIC selection showed that primary sites, T, N, M classification, LNE, LNR and age were retained as significant factors in 86.81%, 100%, 96.05%, 100%, 99.93%, 100% and 100% of the random samples.

**Table 1:** Characteristics of SEER patients with lymph node (LN) positive salivary gland cancer

Categorical variables	No. of patients (*N* = 1210)	%
Race		
White	1042	86.1
Black	71	5.9
Other	97	8.0
Gender		
Male	843	69.7
Female	367	30.3
Year of diagnosis		
1988–1994	103	8.5
1995–2001	290	24.0
2002–2008	817	67.5
Primary site		
Parotid	968	80.0
Submandibular	201	16.6
Sublingual	9	0.7
Others	32	2.6
Histologic subtype		
Squamous cell carcinoma	289	19.8
Adenocarcinoma	187	14.6
Adenoid cystic carcinoma	92	7.6
Mucoepidermoid carcinoma	234	19.3
Other	408	38.6
Grade		
I	35	2.9
II	185	15.3
III	499	41.2
IV	237	19.6
Unknown	254	21.0
Surgery and radiation		
Both	930	76.9
No	280	23.1
T staging		
T1 + T2	355	29.3
T3 + T4	744	61.5
Tx	111	9.2
N staging		
N1	456	37.7
N2	725	59.9
N3	29	2.4
M staging		
M0	1124	92.9
M1	48	4.0
Mx	38	3.1
Continuos variables	Median (range)	
Age	65 years (6–100)	
No. of LN examined	16 (1–90)	
No. of positive LNs	2 (1–83)	
Lymph node ratio	0.31 (0.01–1.00)

**Table 2:** Prognostic factors of SEER cause-specific survival among SEER patients with lymph node (LN) involved salivary gland cancer

Variables	Hazard ratio (95% CIs)	*P*
Primary site		
Parotid (reference)		
Submandibular	1.413 (1.126–1.773)	0.002
Sublingual	1.230 (0.398–3.919)	0.702
Others	0.684 (0.337–1.386)	0.291
T classification		
T1 + T2 (reference)		
T3 + T4	1.870 (1.474–2.373)	< 0.001
Tx	0.937 (0.613–1.431)	0.762
N classification		
N1 (reference)		
N2	1.421 (1.130–1.787)	0.003
N3	2.099 (1.266–3.479)	0.004
M classification		
M0 (reference)		
M1	5.229 (3.604–7.586)	< 0.001
Mx	1.452 (0.846–2.494)	0.176
No. of LNs examined	1.012 (1.007–1.017)	< 0.001
Lymph node ratio(LNR)	2.208 (1.671–2.918)	< 0.001
Age	1.017 (1.007–1.017)	< 0.001

### Cutoff points identification of LNR

In the second stage of cutoff point identification, a linear trend between LNR and SGC mortality was found by spline smoothing, and the upper and lower tertile points of LNR were 0.17 and 0.55, which were defined as the first pair of cutoff points. The X-tile program then identified 0.17/0.56 as the second pair of cutoff points. Using cutoff points of 0.17/0.55 and 0.17/0.56, we classified patients as R1, R2 and R3 three risk groups, respectively. Table 3 summarizes the univariate Log-rank *χ*^2^ test, and Kaplan–Meier survival estimates according to risk group defined by specific LNR categories. Using the multivariate model identified in Table 2, the predictive accuracy of categorical LNR were compared with continuous LNR by the *C*-index and AIC value. As listed in Table 3, the cutoff points 0.17/0.56 showed a homogeneous patient grouping, the largest Log-rank *χ*^2^ value, the highest *C*-index and smallest AIC value, which indicated high statistical significance representing the optimum prognostic stratification and predictive accuracy. Therefore, cutoff points of 0.17/0.56 were used for further analysis.

**Table 3:** Univariate and multivariate analysis of categorical and continuous LNR with SEER cause-specific survival (CSS) of SEER salivary gland cancer pN+patients

LNR classification	Number	5-year	Log-rank	Multivariate analysis^a^		
		CSS(%)	*χ*^2^ (*P* value)	HR (95% CI)	*C*-index	AIC
Cutpoints 0.17/0.55			32.149		0.695	5626.36
R1: 0–0.17	430	57.2	(< 0.001)	Reference		
R2: 0.17–0.55	380	52.9		1.284 (0.993–1.663)		
R3: > 0.55	400	39.9		1.986 (1.560–2.528)		
Cutpoints 0.17/0.56			33.062		0.696	5625.15
R1: 0–0.17	430	57.2	(< 0.001)	Reference		
R2: 0.17–0.56	384	53.1		1.276 (0.986–1.650)		
R3: > 0.56	396	39.7		2.008 (1.578–2.557)		
Continuous LNR				2.208 (1.671–2.918)	0.692	5627.78

^a^The multivariate analysis was adjusted using the same Cox regression model at Table 2

### R classification as a complementary system to TNM staging

**Fig. 1:**
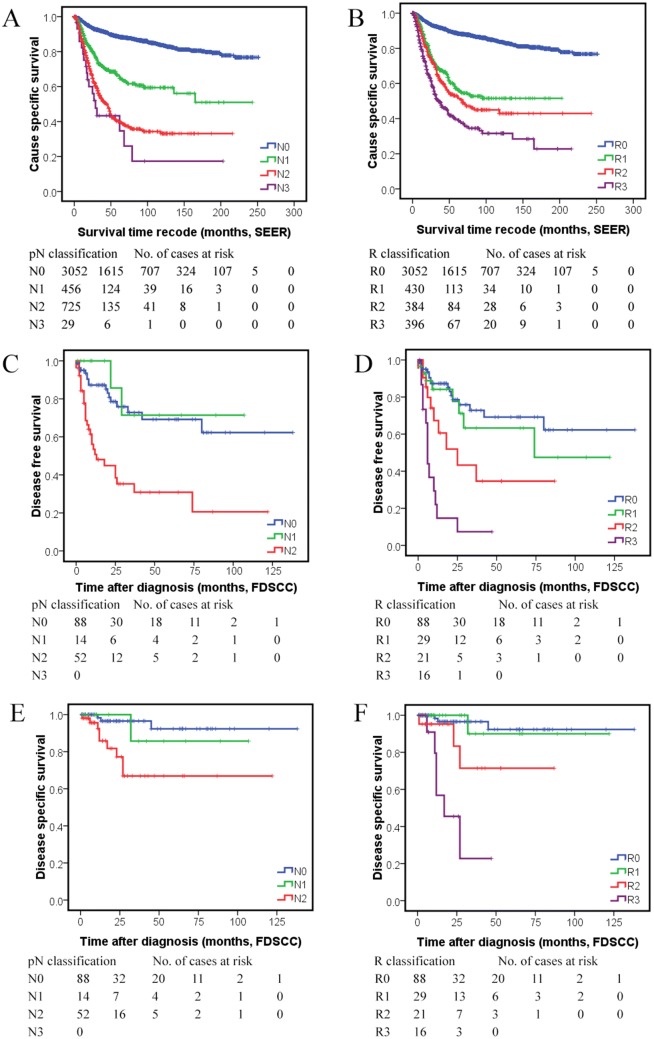
Kaplan–Meier survival estimates according to pN classification and R classification: cause-specific survival (CSS) of the SEER set with different pN classification (**a**) and R classification (**b**); disease-free survival (DFS) of the FUSCC set with different pN classification (**c**) and R classification (**d**); disease-specific survival (DSS) of the FUSCC set with different pN classification (**e**) and R classification (**f**); compared with the overlap of the pN classification survival curves, R classification showed better discriminatory ability for salivary gland cancer survival

Using cutoff points 0.17/0.56, we then classified SEER patients with detailed pN staging (*N* = 4262) as R0-3 four risk groups. Compared with the imbalance of pN2 and pN3 CSS curves, the R classification CSS curves of the SEER set were clearly separated without cross (Fig. 1a, b). R classification also showed a higher *C*-index and lower AIC value than N classification (Table 4) in the multivariate Cox regression model with either R classification or N classification and variables identified by univariate analysis as covariates (Supplement Table 2). For 3944 patients with detailed TNM staging information, when the Cox regression model enrolled variables associated with CSS (Supplement Table 3) and TNM, TRM or TNM + R staging were compared, TNM + R staging showed the highest *C*-index and lowest AIC value which suggested that TNM + R staging had the best predictive accuracy, which followed by TRM staging (Table 4).

**Table 4:** Different staging systems for SEER cause-specific survival (CSS) of SEER patients with salivary gland cancer

Staging system	Patients no.^a^	5-year CSS survival (%)	Log-rank *χ*^2^ (*P* value)	Multivariate analysis^b^		
				HR (95% CI)	*C*-index	AIC
N classification			755.366		0.830	11,304.72
N0	3052	88.6	(< 0.001)	Reference		
N1	456	66.0		1.866 (1.500–2.322)		
N2	725	40.2		2.643 (2.197–3.179)		
N3	29	43.3		4.381 (2.685–7.149)		
R classification			722.815		0.832	11,287.32
R0	3052	88.6	(< 0.001)	Reference		
R1: 0–0.17	430	57.2		1.703 (1.363–2.127)		
R2: 0.17–0.56	384	53.1		2.215 (1.789–2.742)		
R3: > 0.56	396	39.7		3.348 (2.738–4.095)		
TNM staging			964.290		0.823	10,583.18
I	1069	96.6	(< 0.001)	Reference		
II	753	93.2		1.788 (1.163–2.749)		
III	908	79.1		4.601 (3.188–6.640)		
IVA	990	52.5		8.457 (5.868–12.189)		
IVB	131	42.3		12.999 (8.477–19.934)		
IVC	93	17.6		26.937 (17.476–41.518)		
TRM staging^c^			1000.667		0.828	10,551.61
I	1069	96.6	(< 0.001)			
II	753	93.2		1.784 (1.161–2.743)		
III	890	77.1		4.428 (3.066–6.395)		
IVA	725	61.1		7.037 (4.859–10.191)		
IVB	414	40.6		12.397 (8.519–18.041)		
IVC	93	17.6		26.557 (17.246–40.895)		
TNM + R staging					0.830	10,536.22

^a^For N classification and R classification, a total of 4262 patients with the number of lymph nodes examined were analyzed. For TNM staging, TRM staging, TNM + R staging, a total of 3944 patients were enrolled for sufficient TNM staging information

^b^N classification and R classification were adjusted for primary site, histologic type, histologic grade, site-directed surgery, radiotherapy, the number of lymph nodes examined, T classification, M classification and age of the SEER patients (variables identified in Supplement Table 2). TNM staging, TRM staging and TNM + R staging (TNM staging and R classification as two variables enrolled in the Cox regression together) were adjusted for primary site, histologic type, histological grade, site directed surgery, radiation, the number of lymph nodes examined and age of the SEER patients (variables identified in Supplement Table 3)

^c^TRM staging system was defined as I: T1-R0-M0; II: T2-R0-M0; III: T3-R0-M0, T1-R1-M0, T2-R1-M0, T3-R1-M0; IVA: T4a-R0-M0, T4a-R1-M0, T1-R2-M0, T2-R2-M0, T3-R2-M0, T4a-R2-M0; IVB: T4b-Any R-M0, Any T-R3-M0; IVC: any T-Any R-M

### Validation of R classification in FUSCC patient set

To validate LNR staging in the FUSCC patient set, 66 pN+ cases were analyzed and the median number of LNE, positive LNs and LNR were 25 (range 4–86), 4 (range 1–75) and 0.250 (range 0.026–1). The multivariate Cox regression model confirmed that continuous LNR [hazard ratio (HR) 10.503; 95% confidence interval (CI) 3.084–35.770] and postoperative radiation (HR 0.406; 95% CI 0.185–0.892) were significant prognostic factors for DFS (disease-free survival) in the FUSCC patients with pN+. The Cox regression model also identified that LNR (HR 15.72; 95% CI 2.364–104.5) was the only significant prognostic factor for DSS in these patients. When R classification (categorical LNR using cutoff points 0.17/0.56) identified in the SEER set were compared with continuous LNR in predicting FUSCC patient DSS, the AIC value and *C*-index were 60.32 vs 65.55, and 0.802 vs 0.774, respectively (Supplement Table 4), which suggested that R classification performed better than continuous LNR in predicting DSS in FUSCC patients. For the clinical use of R staging, 154 FUSCC cases with neck dissection were analyzed. The Cox regression model included either N or R classification and variables listed in Supplement Table 5 and 6, and identified R classification as an independent prognostic variable for DFS and DSS in the set (Table 5). Compared with pN classification, R classification showed the better separation of survival curves (Fig. 1c–f), lower AIC values and higher *C*-index for predicting DSS (Table 5). The TNM + R staging system also had superiority over TNM staging in predicting SGC mortality (higher *C*-index and lower AIC values, Table 5).

**Table 5:** Validation of R classification system for predicting salivary gland cancer disease free survival (DFS) and disease specific survival (DSS) in FDSCC patients with neck dissection

Staging	Case No	5-year DFS	Log-rank *χ*^2^ (*P* value)	Multivariate analysis of DFS^a^			5-year DSS	Log-rank *χ*^2^ (*P* value)	Multivariate analysis of DSS^b^		
				HR (95% CI)	*C*-index	AIC			HR (95% CIs)	*C*-index	AIC
N classification			25.096		0.73	400.009		10.799		0.734	106.753
N0	88	69.2	(< 0.001)	Reference			92.3	(0.005)	Reference		
N1	14	71.4		0.541 (0.124–2.358)			85.7		1.914(0.199–18.460)		
N2	52	30.9		3.235 (1.746–5.995)			66.9		6.552(1.764–24.330)		
N3	0										
R classification			43.897		0.72	397.649		37.986		0.796	98.113
R0	88	69.2	(< 0.001)	Reference			92.3	(< 0.001)	Reference		
R1: 0–0.17	29	63.3		1.249 (0.535–2.918)			90.0		1.027(0.107–9.891)		
R2: 0.17–0.56	21	34.6		2.527 (1.137–5.616)			71.4		4.715(0.949–23.429)		
R3: > 0.56	16	0		7.007 (3.239–15.156)			0		21.677(5.285–88.916)		
TNM staging ^c^					0.663	412.414				0.736	110.621
TNM + R staging^d^					0.728	402.68				0.856	103.149

^a^N classification and R classification were adjusted for extraparenchymal invasion

^b^N classification or R classification was the only prognostic variable kept in the Cox model

^c^TNM staging was the only variable in the Cox regression model

^d^Both TNM staging and R classification were variables in the Cox regression model

### R classification as an adverse factor for postoperative radiotherapy

**Fig. 2:**
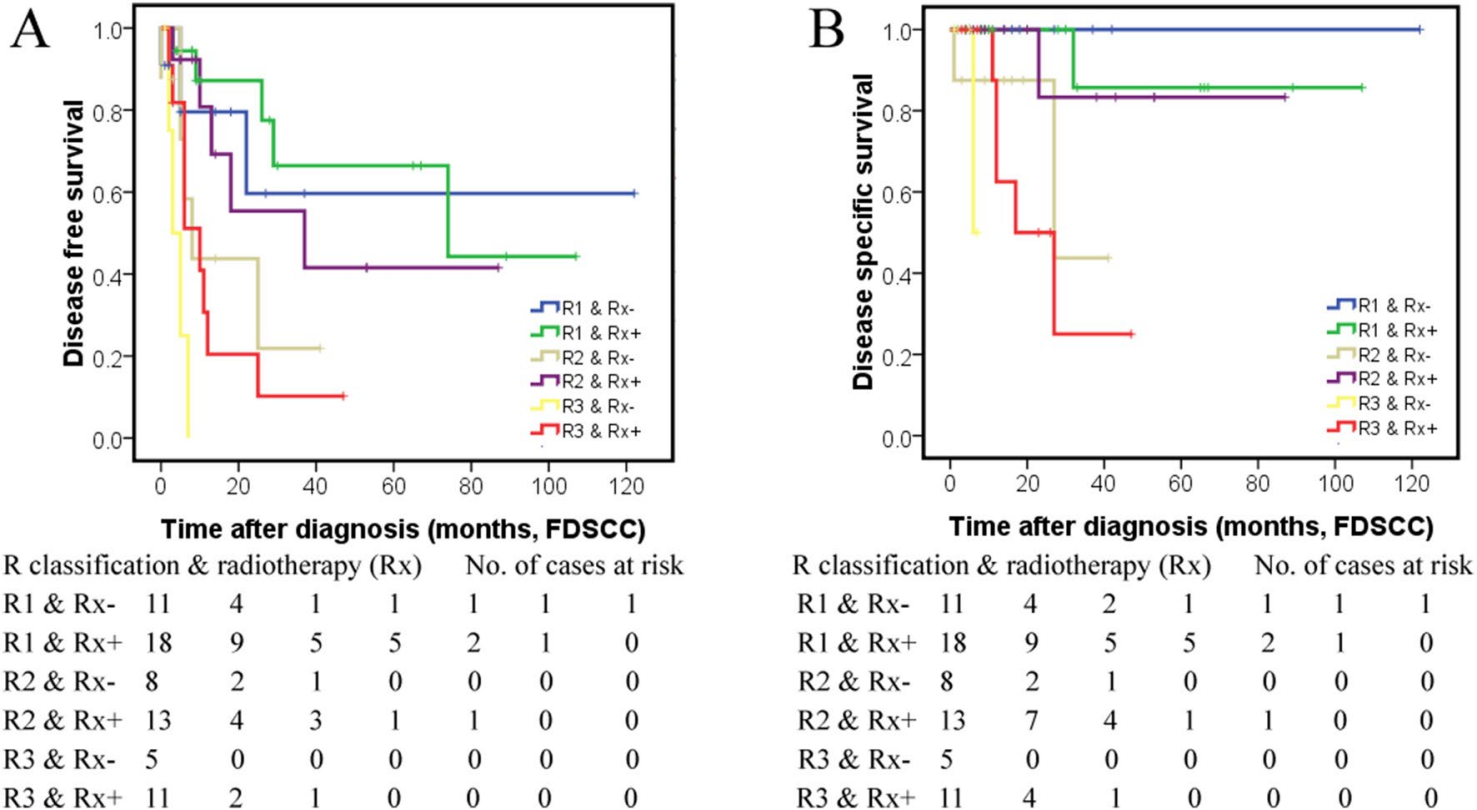
Survival differences of individual R classification FUSCC patients with and without postoperative radiotherapy (Rx): disease-free survival (**a**) and disease-specific survival (**b**)

Radiotherapy is recommended for pN+ SGC cases in the NCCN guidelines [7], while survival benefits from radiotherapy were not observed for both SEER patients and FUSCC patients using pN classification as stratum (Supplement Table 7). For the FUSCC set, the 3-year DFS (Fig. 2a) for R1–3 patients without and with postoperative radiotherapy were 59.7%, 21.9%, 0% and 66.4%, 55.4%, 10.2%, respectively (Log-rank *χ*^2^ 4.733, *P* = 0.030). The 3-year DSS (Fig. 2b) for R1-3 FUSCC patients without and with radiotherapy were 100%, 43.8%, 0% and 85.7%, 83.3%, 25.0%, respectively (Log-rank *χ*^2^ 2.601, *P* = 0.107). Significant survival improvements were observed for R2 and R3 FUSCC patients with postoperative radiotherapy.

## Discussion

Extensive studies have demonstrated that LNR is superior to pN classification in predicting patient prognosis in breast, gastric, and colorectal cancer [8–10, 14]. To our best knowledge, this is the first paper to discuss the role of LNR in SGCs staging. Our analysis revealed that LNR is one of the most important prognostic factors of SGCs (retained as significant prognostic factors in 100% of 10,000 bootstrap resamplings). The discriminative ability of Kaplan–Meier curves (Fig. 1), the smaller AIC and the larger *C*-index values of Cox regression models (Tables 4, 5) all support that R classification and TNM + R staging system have higher predictive accuracy of survival than N classification and TNM staging, respectively [16, 19]. According to our findings, LNR staging was a better predictor for SGC survival and should thus be used to complement TNM staging system.

Although the categorization of a continuous covariate (LNR) discards data and introduces a measurement error, it indeed brings simplicity and is therefore preferred in daily clinical practice [8]. Appropriate cutoff points are critical in the categorization of LNR because they provide consistent groupings between studies and ensure that each group contains an adequate number of individuals and events [17]. In this paper, we adopted two pairs of cutoff points for LNR. One was fixed centiles, to be more specific, tertiles. The other pair was calculated by the X-tile program, which uses the minimum *P* values from Log-rank statistics that control the inflated type I error and minimize the loss of information from multiple testing through cross-validation [9, 18]. When compared with continuous LNR, the regression model with the categorical LNR (both cutoff points) showed lower AIC and higher *C*-index values, supporting that R classification does not decrease the statistical power for survival prediction. We recommend cutoff points 0.17/0.56 identified by X-tile for further validation and clinical usage because of the homogeneous patient grouping, the lowest AIC and highest *C*-index value in both patient sets. However, the issue of optimal cutoffs remains open and the cutoffs based on individual dataset need further validation.

Identification of high-risk patients and selecting patients for postoperative therapy are two major clinically important requirements of a novel staging system. Compared with N3 classification of SEER cases (0.68%, 29/4262), the percent of R3 patients was 9.29% (396/4262), while the 5-year CSS decreased from 43.3% to 39.7%. The shift in staging was also confirmed in the FUSCC set without pN3 patients, while 10.39% of FUSCC patients were identified as R3, and the 5-year DFS and DSS were all 0%. Postoperative radiation is recommended for all pN+ cases in SGC [7]. Concurrent chemoradiotherapy has also been reported to result in excellent local control in a subgroup of SGC patients with adverse prognostic factors [20]. However, no survival benefits were observed for specific N classification cases with and without radiation in both the SEER and FUSCC set. As presented in the Results and Fig. 2, no survival benefit was achieved in R1 FUSCC patients. For R2 patients, both DFS and DSS improvement from postoperative radiotherapy were observed. For R3 patients, although significantly improved 3-year DSS (0–10.2%) and DFS (0–25%) were observed, the survival rates were still low, suggesting the need for more intensified therapy. Compared with N classification, R classification shows superiority for selecting high-risk patients and better predictive ability of treatment benefit. R classification-based analysis and design of clinical trials deserve further research.

The strength of the present study draws on the complementary data collection system and the cross-validation of the SEER and FUSCC datasets. SEER data are extracted retrospectively from registries comprising 26% of the US population, which is considered representative of the entire population, and selection bias, recall bias, treatment fads, the influence of loss to follow-up and other oversights associated with a single institution’s data collection were minimized [14, 15]. Nevertheless, the inter-institution differences in patient management, unrecorded details of pathologic reports and covariates may compromise the outcome analysis in the SEER data. The FUSCC data was from a single-center, with the same team of oncologists and pathologists managing all patients, and all the potential covariates were analyzed in the current study. There is no generally accepted guidance for the postoperative management of SGC. Nevertheless, an appropriate postoperative follow-up strategy for SGC patients is critical to both the management of the disease and the accuracy of survival data. De Felice et al. recommend a routine combination of complete head and neck exam and diagnostic imaging exams (DW-MRI imaging and/or CT with contrast) [2]. In our institution, apart from physical examination and a head MRI imaging, the SGC patients also receive a neck ultrasound exam for the early detection of suspicious lymph nodes. While the patient series in one institution is small and the results only represent that center’s experience, the current study validated the LNR staging system in two independent patient sets and showed the valuable predictive ability of LNR as a prognostic factor.

Although the LNR is superior to the N classification for prognostic staging, limitations still exist for R classification. It could only be used for postoperative staging, and the number of nodes harvested is an important factor for LN ratio. As a significant prognostic factor for the SEER dataset (Table 2), LNE was rejected by multivariate analysis in the FUSCC dataset (Table 5). One of the possible explanations is that the standardized anatomic compartments and levels-based LN dissection and pathologic review with at least 4 LNs examined in one high disease volume hospital could lower the effect of the LNE in the prognostic model. Therefore, compartment-based neck dissection and sufficient LNs examination may still be necessary for accurate LNR staging of SGCs.

## Conclusion

We clearly identified that the LNR was an independent prognostic factor of SGC, and R classification (LNR = 0, LNR = 0–0.17, LNR = 0.17–0.56 and LNR > 0.56) defines SGC mortality adequately. R classification is complementary to the TNM staging system. R classification-based stratification of patients for postoperative therapy and clinical trials deserves further research.

## Electronic supplementary material

Below is the link to the electronic supplementary material.

Supplementary file1 (DOCX 12 kb)

Supplementary file2 (DOCX 13 kb)

Supplementary file3 (DOCX 13 kb)

Supplementary file4 (DOCX 12 kb)

Supplementary file5 (DOCX 13 kb)

Supplementary file6 (DOCX 12 kb)

Supplementary file7 (DOCX 14 kb)
