# Supplementary material for: Lymph node ratio (LNR) as a complementary staging system to TNM staging in salivary gland cancer
Source: Eur Arch Otorhinolaryngol. 2019 Sep 11;276(12):3425–34. doi: 10.1007/s00405-019-05597-0 (PMC6858905; doi:10.1007/s00405-019-05597-0)
Supplement: Supplementary file 1 — Supplementary file1 (DOCX 12 kb) [file 405_2019_5597_MOESM1_ESM.docx]

**Supplement Table 1** Univariate analysis of SEER cause specific survival (CSS) of SEER salivary gland cancer cases with lymph nodes metastasis (*N*=1210)

| Variables | 5 year CSS | Log-rank χ^2^ | *P* value |
| --- | --- | --- | --- |
| **Primary site**  Parotid  Submandibular  Sublingual  Others  **Histologic subtype**  Squamous cell carcinoma  Adenocarcinoma NOS  Adenoid cystic carcinoma  Mucoepidermoid carcinoma  Other  **Grade**  I  II  III  IV  Unknown  **T classification**  T1+T2  T3+T4  Unknown  **N classification**  N1  N2  N3  **M classification**  M0  M1  Unknown | 52.9  37.2  64.3  60.8  52.6  39.0  43.7  53.6  55.1  83.5  60.9  43.6  45.7  54.1  66.5  41.8  54.1  66.7  40.2  43.3  52.2  9.5  46.4 | 12.509  12.317  18.683  43.626  45.098  102.744 | 0.006  0.015  0.001  < 0.001  < 0.001  <0.001 |
