# Supplementary material for: Lymph node ratio (LNR) as a complementary staging system to TNM staging in salivary gland cancer
Source: Eur Arch Otorhinolaryngol. 2019 Sep 11;276(12):3425–34. doi: 10.1007/s00405-019-05597-0 (PMC6858905; doi:10.1007/s00405-019-05597-0)
Supplement: Supplementary file 2 — Supplementary file2 (DOCX 13 kb) [file 405_2019_5597_MOESM2_ESM.docx]

**Supplement Table 2** Univariate analysis of SEER cause specific survival (CSS) of SEER salivary gland cancer cases with detailed N staging information (N = 4262)

| Variables | N | 5 year CSS | Log-rank χ^2^ | *P* value |
| --- | --- | --- | --- | --- |
| **Primary site**  Parotid  Submandibular  Sublingual  Others  **Histologic subtype**  Squamous cell carcinoma  Adenocarcinoma NOS  Adenoid cystic carcinoma  Mucoepidermoid carcinoma  Other  **Grade**  I  II  III  IV  Unknown  **T classification**  T1+T2  T3+T4  Unknown  **M classification**  M0  M1  Unknown  **N classification**  N0  N1  N2  N3  **R classification**  R0  R1: 0-0.17  R2: 0.17-0.56  R3: > 0.56  **Surgery and radiation**  Both  No  **Site directed surgery**  Yes  No | 3523  624  39  76  672  428  555  1083  1524  425  1025  937  495  1380  2188  1736  338  4100  93  69  3052  456  725  29  3052  430  384  396  2567  1695  4206  56 | 80.3  70.7  85.0  75.7  63.5  66.8  80.1  86.2  82.5  95.1  87.2  57.3  62.3  86.1  91.1  63.0  76.0  80.4  17.6  58.6  88.6  66.0  40.2  43.3  88.6  57.2  53.1  39.7  73.1  87.3  79.2  47.3 | 27.085  177.394  393.335  412.813  320.874  755.366  722.815  105.172  43.603 | < 0.001  < 0.001  < 0.001  < 0.001  < 0.001  < 0.001  < 0.001  < 0.001  < 0.001 |
