# Supplementary material for: Lymph node ratio (LNR) as a complementary staging system to TNM staging in salivary gland cancer
Source: Eur Arch Otorhinolaryngol. 2019 Sep 11;276(12):3425–34. doi: 10.1007/s00405-019-05597-0 (PMC6858905; doi:10.1007/s00405-019-05597-0)
Supplement: Supplementary file 3 — Supplementary file3 (DOCX 13 kb) [file 405_2019_5597_MOESM3_ESM.docx]

**Supplement Table 3** Univariate analysis of SEER cause specific survival (CSS) of SEER salivary gland cancer cases with detailed TNM staging information (N = 3944)

| Variables | N | 5-year CSS | Log-rank χ^2^ | *P* value |
| --- | --- | --- | --- | --- |
| **Primary site**  Parotid  Submandibular  Sublingual  Others  **Histologic subtype**  Squamous cell carcinoma  Adenocarcinoma NOS  Adenoid cystic carcinoma  Mucoepidermoid carcinoma  Other  **Grade**  I  II  III  IV  Unknown  **Surgery and radiation**  Both  No  **Site directed surgery**  Yes  No  **R staging**  R0  R1  R2  R3  **TNM staging**  I  II  III  IVA  IVB  IVC  **TRM staging**  I  II  III  IVA  IVB  IVC | 3280  569  35  60  618  401  517  1002  1406  399  949  882  472  1242  2438  1506  3909  35  2835  402  363  344  1069  753  908  990  131  93  1069  753  890  725  414  93 | 79.9  69.5  84.4  75.1  61.9  65.9  79.7  85.6  82.5  95.6  86.7  56.7  61.8  85.7  72.8  87.0  78.6  41.7  88.2  56.7  53.0  36.7  96.6  93.2  79.1  52.5  42.3  17.6  96.6  93.2  77.1  61.1  40.6  17.6 | 26.109  176.086  372.234  91.266  54.986  699.659  964.290  1000.667 | < 0.001  < 0.001  < 0.001  < 0.001  < 0.001  < 0.001  < 0.001  < 0.001 |
