# Supplementary material for: Lymph node ratio (LNR) as a complementary staging system to TNM staging in salivary gland cancer
Source: Eur Arch Otorhinolaryngol. 2019 Sep 11;276(12):3425–34. doi: 10.1007/s00405-019-05597-0 (PMC6858905; doi:10.1007/s00405-019-05597-0)
Supplement: Supplementary file 4 — Supplementary file4 (DOCX 12 kb) [file 405_2019_5597_MOESM4_ESM.docx]

**Supplement Table 4** Multivariate analysis of continuous LNR and categorical LNR (R classification) in predicting disease-free survival (DFS) and disease-specific survival (DSS) of FDSCC patients with lymph node metastasis

| Patients’ variables | N | Multivariate analysis of DFS‡ | | |  | Multivariate analysis of DSS† | | |
| --- | --- | --- | --- | --- | --- | --- | --- | --- |
|  |  | OR (95% CI) | AIC | C-index |  | OR(95% CI) | AIC | C-index |
| **Continuous LNR**  **R classification**  R1: 0-0.17  R2: 0.17-0.56  R3: > 0.56 | 29  21  16 | 10.503(3.084-35.770)  Reference  2.133(0.832-5.467)  7.852(2.998-20.565) | 208.46  204.93 | 0.727  0.73 |  | 15.72(2.364-104.5)  Reference  4.66(0.483-44.93)  21.95(2.551-188.92) | 65.55  60.32 | 0.774  0.802 |

‡ Continuous LNR or R classification was adjusted for postoperative radiation. † Continuous LNR or R classification was the only variable kept in the Cox regression model.
