# Supplementary material for: Lymph node ratio (LNR) as a complementary staging system to TNM staging in salivary gland cancer
Source: Eur Arch Otorhinolaryngol. 2019 Sep 11;276(12):3425–34. doi: 10.1007/s00405-019-05597-0 (PMC6858905; doi:10.1007/s00405-019-05597-0)
Supplement: Supplementary file 5 — Supplementary file5 (DOCX 13 kb) [file 405_2019_5597_MOESM5_ESM.docx]

**Supplement Table 5** Univariate analysis of clinicopathologic variables associated with salivary gland cancer disease free survival (DFS) of FDSCC set

| Patients’ variables | N | 5-year DFS (%) | Log-rank χ^2^ value | *P* value |
| --- | --- | --- | --- | --- |
| **Gender**  Male  Female  **Extraparenchymal extension**  No  Yes  **Grade**  Low  Medium  High  **Tumor size**  ≤ 2 cm  2-4 cm  > 4cm  **Lymphatic/vascular invasion**  No  Yes  **Extracapsular invasion**  No  Yes  **T classification**  T1  T2  T3  T4  **N classification**  N0  N1  N2 | 84  70  70  84  36  46  72  54  79  21  135  19  118  36  33  38  40  43  88  14  52 | 48.8  68.1  70.2  44.3  91.1  55.5  41.9  85.3  41.5  38.2  59.3  24.9  60.9  41.0  92.2  47.0  45.3  50.03  69.2  71.4  30.9 | 4.205  8.794  10.982  9.798  4.806  4.485  8.130  25.096 | 0.040  0.003  0.001  0.007  0.028  0.034  0.043  < 0.001 |
