# Supplementary material for: Lymph node ratio (LNR) as a complementary staging system to TNM staging in salivary gland cancer
Source: Eur Arch Otorhinolaryngol. 2019 Sep 11;276(12):3425–34. doi: 10.1007/s00405-019-05597-0 (PMC6858905; doi:10.1007/s00405-019-05597-0)
Supplement: Supplementary file 6 — Supplementary file6 (DOCX 12 kb) [file 405_2019_5597_MOESM6_ESM.docx]

**Supplement Table 6** Univariate analysis of clinicopathologic variables associated with salivary gland cancer disease specific survival (DSS) of FDSCC set

| Patients’ variables | N | 5-year DSS (%) | Log-rank χ^2^ | *P* value |
| --- | --- | --- | --- | --- |
| **Gender**  Male  Female  **Grade**  Low  Medium  High  **Lymphatic/vascular invasion**  No  Yes  **Extracapsular invasion**  No  Yes  **N classification**  N0  N1  N2 | 84  70  36  46  72  135  19  118  36  88  14  52 | 71.0  96.0  100  84.3  74.1  84.2  72.6  89.0  67.9  92.3  85.7  66.9 | 5.675  5.729  3.730  6.719  10.799 | 0.017  0.057  0.053  0.010  0.005 |
