# Supplementary material for: Lymph node ratio (LNR) as a complementary staging system to TNM staging in salivary gland cancer
Source: Eur Arch Otorhinolaryngol. 2019 Sep 11;276(12):3425–34. doi: 10.1007/s00405-019-05597-0 (PMC6858905; doi:10.1007/s00405-019-05597-0)
Supplement: Supplementary file 7 — Supplementary file7 (DOCX 14 kb) [file 405_2019_5597_MOESM7_ESM.docx]

**Supplement Table 7** Survival differences of individual N classification or R classification with and without radiotherapy of SEER pN+ patients and FDSCC pN+ patients

| Patients’ classification | No radiotherapy | |  | With radiotherapy | | Log-rank χ^2^ | *P* value |
| --- | --- | --- | --- | --- | --- | --- | --- |
|  | N | Survival |  | N | Survival |  |  |
| **SEER patients for 5-year Cause specific survival**  **N classification**  N1  N2  N3  **R classification**  R1  R2  R3  **FDSCC patients for 5-year**  **Disease free survival**  **N classification**  N1  N2  N3  **R classification**  R1  R2  R3  **FDSCC patients for 5-year Disease specific survival**  **N classification**  N1  N2  N3  **R classification**  R1  R2  R3 | 139  134  7  96  71  113  6  18  0  11  8  5  6  18  0  11  8  5 | 59.9%  37.9%  57.1%  54.8%  50.6%  43.9%  0%  28.7%  59.7%  0%  0%  100%  64.9%  100%  0%  0% |  | 317  591  22  334  313  283  8  34  0  18  13  11  8  34  0  18  13  11 | 69.4%  40.7%  39.2%  57.9%  53.5%  38.4%  80%  31.5%  66.4%  41.5%  0%  80%  67.4%  85.7%  83.3%  0% | 2.200  0.204  1.801  4.733  0.009  2.601 | 0.138  0.651  0.180  0.030  0.926  0.107 |
